# Supplementary material for: UDP-glucuronate metabolism controls RIPK1-driven liver damage in nonalcoholic steatohepatitis
Source: Nat Commun. 2023 May 11;14:2715. doi: 10.1038/s41467-023-38371-2 (PMC10175487; doi:10.1038/s41467-023-38371-2)
Supplement: Supplementary file 1 — Supplementary Information [file 41467_2023_38371_MOESM1_ESM.pdf]

SUPPLEMENTARY INFORMATION

Title:

UDP-glucuronate metabolism controls RIPK1-driven liver damage in nonalcoholic steatohepatitis

Author list:

Tao Zhang [1,2,3,9], Na Zhang [4,5,9], Jing Xing [6,9], Shuhua Zhang [1,2,9], Yulu Chen [4,9], Daichao Xu [4,7] & Jinyang Gu [1,2,8*].

Affiliations:

[1] Center for Liver Transplantation, Union Hospital, Tongji Medical College, Huazhong University of Science and Technology, Wuhan, 430022, China.

[2] Key Laboratory of Organ Transplantation, Ministry of Education; NHC Key Laboratory of Organ Transplantation; Key Laboratory of Organ Transplantation, Chinese Academy of Medical Sciences, Wuhan, China.

[3]Division of Endocrinology, Boston Children's Hospital, Harvard Medical School, Boston, MA 02115, USA.

[4]Interdisciplinary Research Center on Biology and Chemistry, Shanghai Institute of Organic Chemistry, Chinese Academy of Sciences, 100 Haike Road, Pudong, Shanghai, 201210, China.

[5]University of Chinese Academy of Sciences, 80 Zhongguancun East St, Haidian, Beijing, 101408, China.

[6]Lingang Laboratory, Xuhui, Shanghai, 200031, China.

[7]Shanghai Key Laboratory of Aging Studies, Shanghai, China.

[8]Department of Transplantation, Xinhua Hospital Affiliated to Shanghai Jiao Tong University School of Medicine, Shanghai, 200092, China.

[9]These authors contributed equally: Tao Zhang, Na Zhang, Jin Xing, Shuhua Zhang, Yulu Chen

*Correspondence to: gjynyd@126.com

Inventory of Supporting Information:

Supplementary Figures 1 to 9

Supplementary Table 1

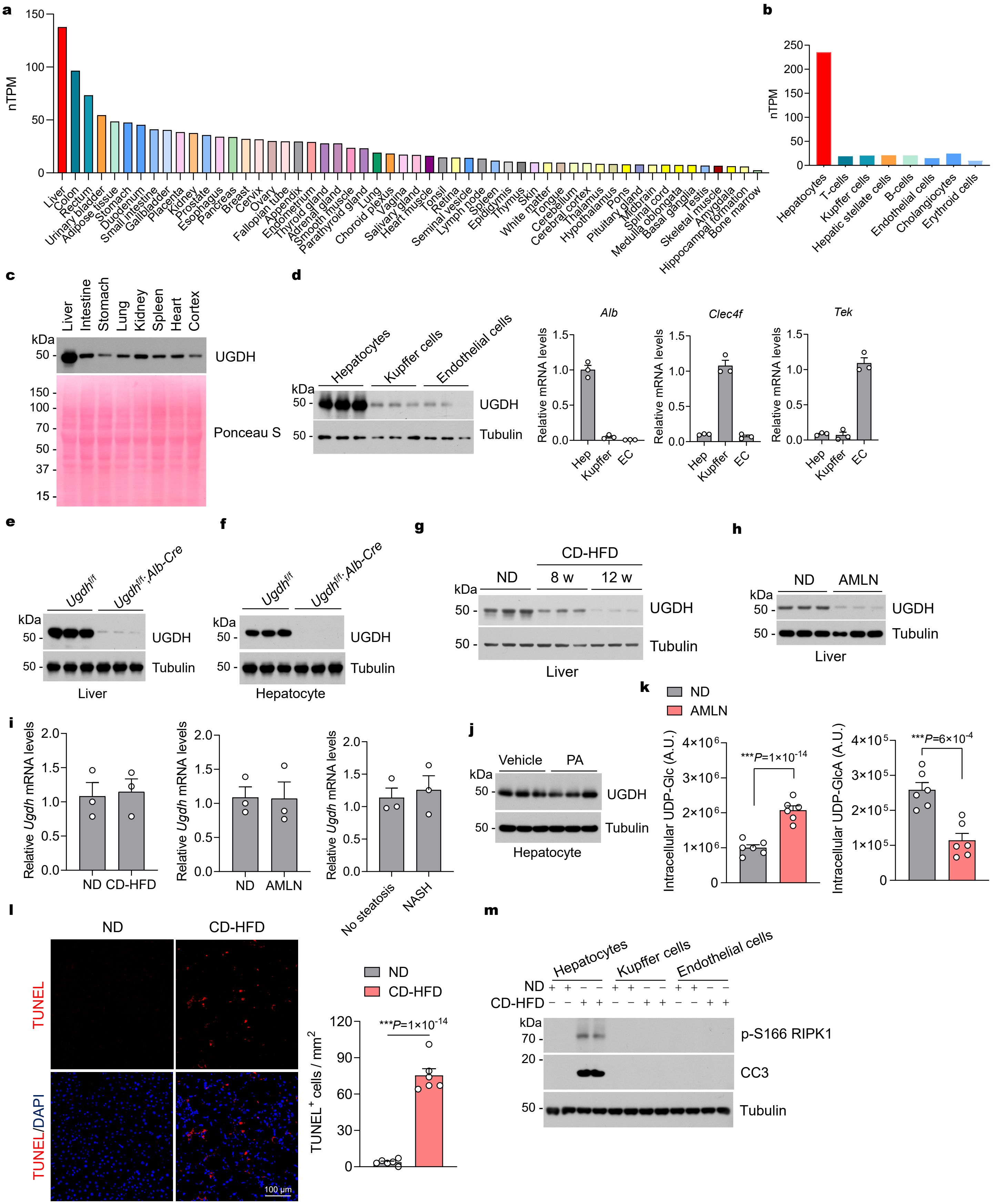

a
nTPM
Liver
Colon
Rectum
Urinary bladder
Adipose tissue
Stomach
Duodenum
Small intestine
Gallbladder
Placenta
Kidney
Prostate
Esophagus
Pancreas
Breast
Cervix
Ovary
Fallopian tube
Appendix
Endometrium
Thyroid gland
Adrenal gland
Smooth muscle
Parathyroid gland
Lung
Choroid plexus
Vagina
Salivary gland
Heart muscle
Tonsil
Retina
Seminal vesicle
Lymph node
Spleen
Epididymis
Thymus
Skin
White matter
Tongue
Cerebellum
Cerebral cortex
Thalamus
Hypothalamus
Pons
Pituitary gland
Midbrain
Spinal cord
Medulla oblongata
Basal ganglia
Testis
Skeletal muscle
Amygdala
Hippocampal formation
Bone marrow
b
nTPM
Hepatocytes
T-cells
Kupffer cells
Hepatic stellate cells
B-cells
Endothelial cells
Cholangiocytes
Erythroid cells
c
Liver
Intestine
Stomach
Lung
Kidney
Spleen
Heart
Cortex
kDa
UGDH
Ponceau S
d
Hepatocytes
Kupffer cells
Endothelial cells
kDa
UGDH
Tubulin
Alb
Clec4f
Tek
Relative mRNA levels
Hep
Kupffer
EC
e
Ugdh^f/f
Ugdh^f/f; Alb-Cre
kDa
UGDH
Tubulin
Liver
f
Ugdh^f/f
Ugdh^f/f; Alb-Cre
kDa
UGDH
Tubulin
Hepatocyte
g
CD-HFD
ND
8 w
12 w
kDa
UGDH
Tubulin
Liver
h
ND
AMLN
kDa
UGDH
Tubulin
Liver
i
Relative Ugdh mRNA levels
ND
CD-HFD
ND
AMLN
No steatosis
NASH
j
Vehicle
PA
kDa
UGDH
Tubulin
Hepatocyte
k
ND
AMLN
Intracellular UDP-Glc (A.U.)
***P=1×10^-14
Intracellular UDP-GlcA (A.U.)
***P=6×10^-4
l
ND
CD-HFD
TUNEL
TUNEL/DAPI
100 μm
ND
CD-HFD
TUNEL+ cells / mm2
***P=1×10^-14
m
Hepatocytes
Kupffer cells
Endothelial cells
ND
CD-HFD
kDa
p-S166 RIPK1
CC3
Tubulin

Supplementary Figure 1. UGDH is highly expressed by hepatocytes and reduced in NASH. **a**, **b**, UGDH is enriched in liver and highly expressed by hepatocytes. The Human Protein Atlas (HPA) RNA-sequencing data of UGDH expression in different tissues (a), hepatocytes and non-parenchymal liver cells (b). **c**, **d**, Representative immunoblotting analysis of UGDH expression in different tissues (c), hepatocytes and non-parenchymal liver cells (d). The purity of isolated each type of cells was determined by measuring the hepatocyte-specific gene *Alb*, Kupffer cell-specific gene *Clec4f*, and endothelia cell-specific gene *Tek*, *n* = 3 mice per group. Each dot represents an individual mouse. Mean ± s.e.m. (d). **e**, **f**, Representative immunoblotting analysis of UGDH expression in livers (e) and primary hepatocytes isolated from livers (f) of 8-week-old mice with indicated genotypes. *n* = 3 mice per group. **g**, **h**, UGDH is reduced in mice fed with CD-HFD (g) or AMLN (h). Representative immunoblotting analysis of UGDH protein levels in the livers of 20-week-old mice fed ND or CD-HFD for indicated time periods (g), and ND or AMLN for 12 weeks (h). *n* = 3 mice per group. **i**, Quantitative RT-PCR analysis of UGDH mRNA levels in livers from 20-week-old mice that were fed ND, CD-HFD, or AMLN for 12 weeks, or from human NASH patients and no steatosis controls. *n* = 3 mice or individuals per group. Each dot represents an individual mouse. Mean ± s.e.m. **j**, Palmitic acid does not induce the reduction of UGDH in hepatocytes. Primary hepatocytes were treated with or without 0.4 mM palmitic acid (PA) for 72 h, then cell lysates were subjected to immunoblotting analysis for UGDH. *n* = 3 independent samples per group. **k**, Intracellular UDP-Glc and UDP-GlcA concentrations in the livers of 20-week-old mice that were fed ND or AMLN for 12 weeks were determined. *n* = 6 mice per group. Mean ± s.e.m. Unpaired two-tailed t-test. **l**, Immunofluorescence images of TUNEL assay on liver sections from 20-week-old mice after feeding with ND or CD-HFD for 12 weeks. Representative images out of *n* = 6 mice for each group are represented. Graph depicting numbers of TUNEL$^+$ cells on liver sections of indicated groups. Each dot represents an individual mouse. Mean ± s.e.m. Unpaired two-tailed t-test. **m**, Representative immunoblotting analysis of p-S166 RIPK1 and CC3 levels in parenchymal and non-parenchymal liver cells from 20-week-old mice after feeding with ND or CD-HFD for 12 weeks. *n* = 2 mice per group.

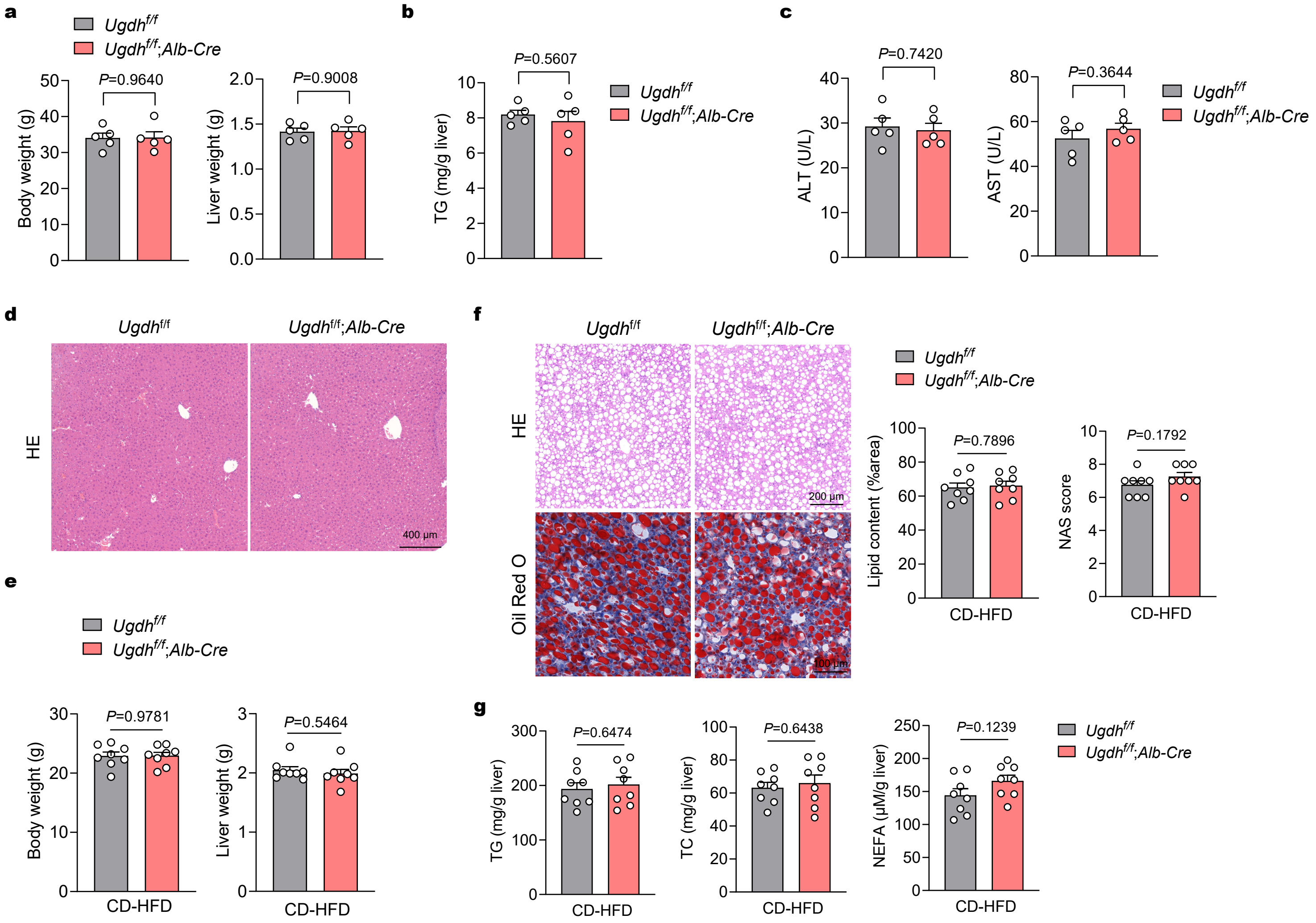

a
Ugdh f/f
Ugdh f/f;Alb-Cre
P=0.9640
Body weight (g)
P=0.9008
Liver weight (g)
b
P=0.5607
TG (mg/g liver)
c
P=0.7420
ALT (U/L)
P=0.3644
AST (U/L)
d
HE
400 μm
e
CD-HFD
P=0.9781
P=0.5464
f
Oil Red O
200 μm
100 μm
P=0.7896
Lipid content (%area)
P=0.1792
NAS score
g
P=0.6474
P=0.6438
TC (mg/g liver)
P=0.1239
NEFA (μM/g liver)

Supplementary Figure 2. Hepatic UGDH knockout does not induce hepatic steatosis in ND and CD-HFD-fed mice.

a-d, Flox and hepatic UGDH-deficient mice were fed with chow diet for 16 weeks. Body weight and liver weight of indicated mice (a). Measurement of liver triglyceride (TG) of indicated mice (b). Serum ALT and AST of indicated mice (c). H&E staining of liver sections of indicated mice (d). *n* = 5 mice for each genotype (a-d). Mean ± s.e.m. Unpaired two-tailed t-test (a-c). **e-g**, Flox and hepatic UGDH-deficient mice (20-week-old) were fed with CD-HFD for 12 weeks. Body weight and liver weight of indicated mice (e). H&E and oil red O staining of liver sections of indicated mice (f). Measurement of liver TG, TC and NEFA of indicated mice (g). *n* = 8 mice for each genotype (e-g). Mean ± s.e.m. Unpaired two-tailed t-test (e-g).

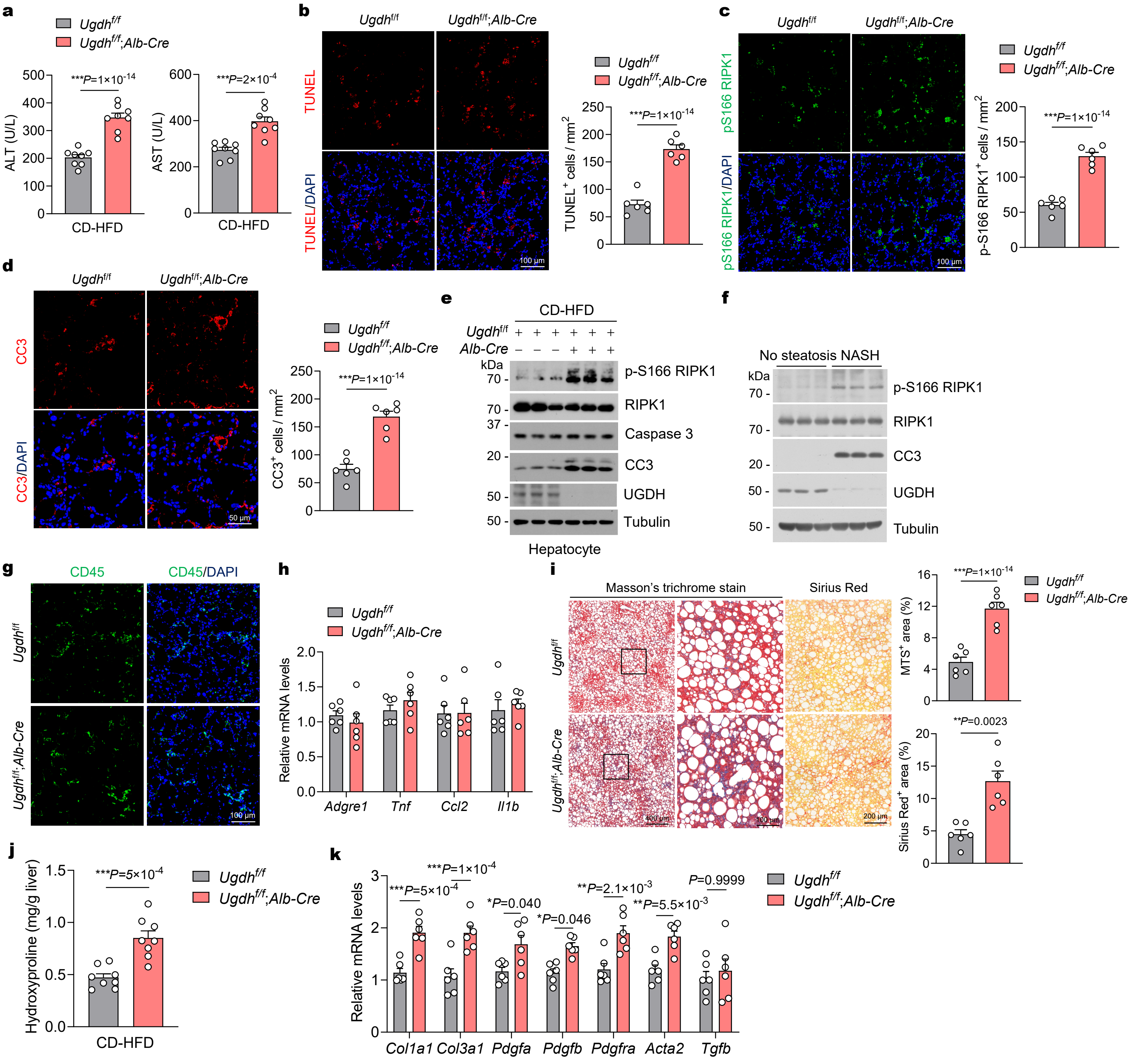
a
Ugdh f/f
Ugdh f/f;Alb-Cre
***P=1×10-14
ALT (U/L)
CD-HFD
***P=2×10-4
AST (U/L)
CD-HFD
b
Ugdhf/f
Ugdhf/f;Alb-Cre
TUNEL
TUNEL/DAPI
100 μm
TUNEL+ cells / mm2
***P=1×10-14
c
pS166 RIPK1
pS166 RIPK1/DAPI
p-S166 RIPK1+ cells / mm2
***P=1×10-14
d
CC3
CC3/DAPI
50 μm
CC3+ cells / mm2
***P=1×10-14
e
CD-HFD
Ugdhf/f
Alb-Cre
kDa
p-S166 RIPK1
RIPK1
Caspase 3
CC3
UGDH
Tubulin
Hepatocyte
f
No steatosis
NASH
kDa
p-S166 RIPK1
RIPK1
CC3
UGDH
Tubulin
g
CD45
CD45/DAPI
100 μm
h
Relative mRNA levels
Adgre1
Tnf
Ccl2
Il1b
i
Masson's trichrome stain
Sirius Red
400 μm
100 μm
200 μm
MTS+ area (%)
***P=1×10-14
Sirius Red+ area (%)
**P=0.0023
j
Hydroxyproline (mg/g liver)
***P=5×10-4
CD-HFD
k
Relative mRNA levels
***P=5×10-4
***P=1×10-4
*P=0.040
*P=0.046
**P=2.1×10-3
**P=5.5×10-3
P=0.9999
Col1a1
Col3a1
Pdgfa
Pdgfb
Pdgfra
Acta2
Tgfb

Supplementary Figure 3. UGDH deficiency promotes liver damage in NASH.

a, Hepatic UGDH deficiency aggravates liver damage in CD-HFD-induced NASH. Serum levels of ALT and AST of 16-week-old mice with indicated genotypes after feeding with CD-HFD for 8 weeks. *n* = 8 mice for each genotype. Each dot represents an individual mouse. Mean ± s.e.m. Unpaired two-tailed t-test. **b**, Hepatic UGDH deficiency promotes cell death in livers of CD-HFD-induced NASH. Immunofluorescence images of TUNEL assay on liver sections from 16-week-old mice with indicated genotypes after feeding with CD-HFD for 8 weeks. Representative images out of *n* = 6 mice for each genotype are represented. Graph depicting numbers of $TUNEL^+$ cells on liver sections of indicated genotypes. Each dot represents an individual mouse. Mean ± s.e.m. Unpaired two-tailed t-test. **c**, **d**, Hepatic UGDH deficiency increases RIPK1 activation (c) and apoptosis (d) in livers of CD-HFD-induced NASH. Immunofluorescence images of p-S166 RIPK1 (c) and CC3 (d) of liver sections from 16-week-old mice with indicated genotypes after feeding with CD-HFD for 8 weeks. Representative images out of *n* = 6 mice for each genotype are represented. Graph depicting numbers of p-S166 $RIPK1^+$ (c) and $CC3^+$ (d) cells on liver sections of indicated genotypes. Each dot represents an individual mouse. Mean ± s.e.m. Unpaired two-tailed t-test. **e**, Hepatic UGDH deficiency promotes RIPK1 activation and apoptosis in hepatocytes from CD-HFD-fed mice. Representative immunoblotting analysis of p-S166 RIPK1 and CC3 protein levels in primary hepatocytes isolated from livers of 16-week-old mice with indicated genotypes after feeding with CD-HFD for 8 weeks. *n* = 3 mice per group. **f**, Representative immunoblotting analysis of p-S166 RIPK1 and CC3 protein levels in human NASH livers and no steatosis controls. *n* = 3 individuals per group. **g**, **h**, Hepatic UGDH deficiency does not affect macrophage infiltration and inflammation in livers of CD-HFD-induced NASH. Immunofluorescence images of CD45 (g) and quantitative RT-PCR analysis of pro-inflammatory cytokines and chemokines (h) of livers from 16-week-old mice with indicated genotypes after feeding with CD-HFD for 8 weeks. Representative images out of *n* = 6 mice for each genotype are represented (g). *n* = 6 mice per genotype. Each dot represents an individual mouse. Mean ± s.e.m (h). **i**, Hepatic UGDH deficiency promotes liver fibrosis in CD-HFD-induced NASH.

Representative images of Masson's trichrome (MTS) and Sirius red stained liver sections of 16-week-old mice with indicated genotypes after feeding with CD-HFD for 8 weeks. Graph depicting percentage of MTS^+ and Sirius red^+ area on liver sections of indicated genotypes. n = 6 mice for each genotype. Each dot represents an individual mouse. Mean ± s.e.m. Unpaired two-tailed t-test. **j**, **k**, Hepatic UGDH deficiency increases liver hydroxyproline and the expression of liver fibrogenic parameters in CD-HFD-induced NASH. Liver hydroxyproline (j) and mRNA expression of fibrogenic parameters (k) in livers of 16-week-old mice with indicated genotypes after feeding with CD-HFD for 8 weeks. n = 6 mice for each genotype. Each dot represents an individual mouse. Mean ± s.e.m. Unpaired two-tailed t-test (j). Two-way ANOVA, post hoc Bonferroni's test (k).

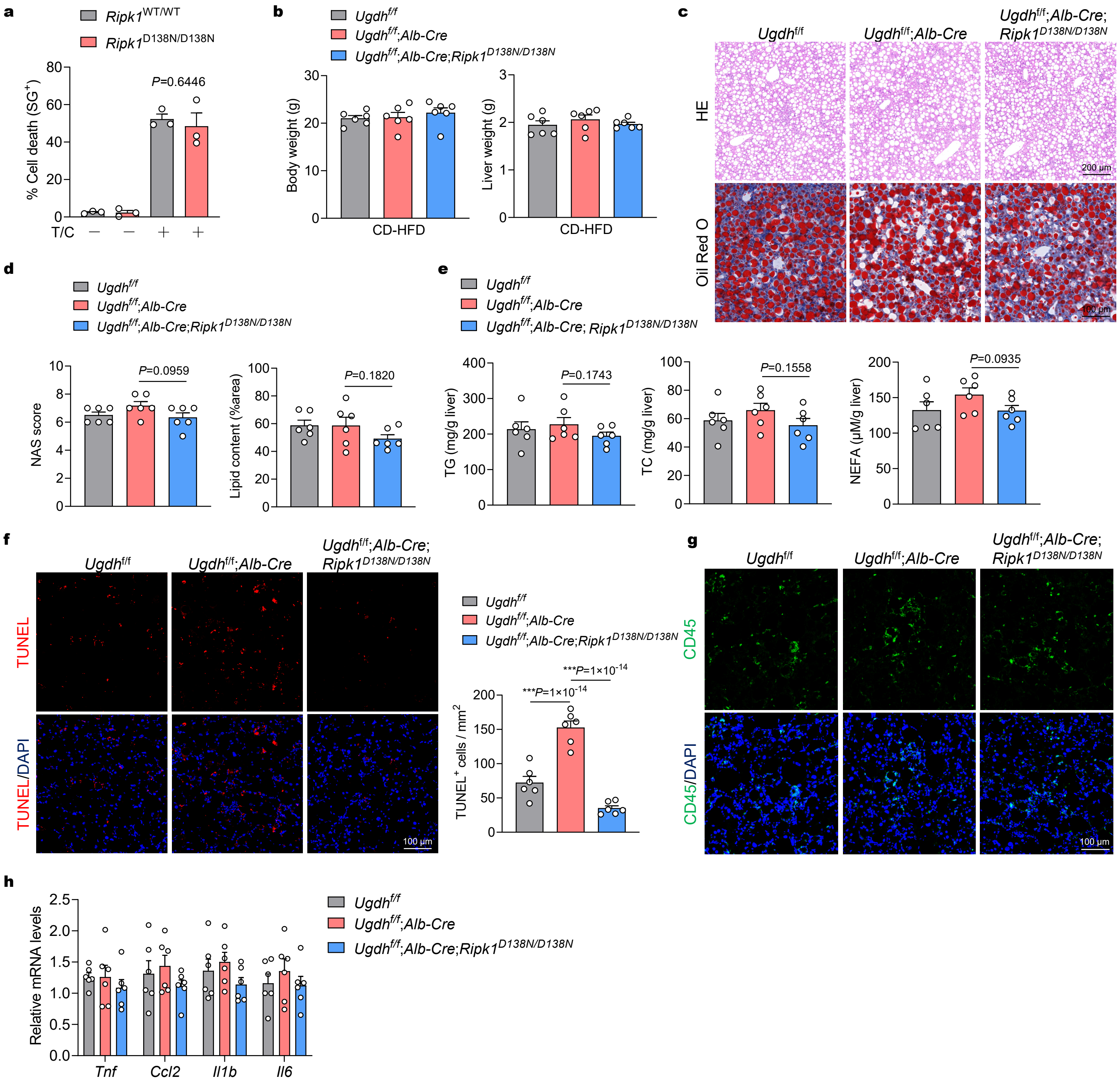
a
Ripk1WT/WT
Ripk1D138N/D138N
% Cell death (SG+)
P=0.6446
T/C
b
Ugdhf/f
Ugdhf/f;Alb-Cre
Ugdhf/f;Alb-Cre;Ripk1D138N/D138N
Body weight (g)
Liver weight (g)
CD-HFD
c
HE
Oil Red O
200 μm
100 μm
d
NAS score
P=0.0959
Lipid content (%area)
P=0.1820
e
TG (mg/g liver)
P=0.1743
TC (mg/g liver)
P=0.1558
NEFA (μM/g liver)
P=0.0935
f
TUNEL
TUNEL/DAPI
100 μm
TUNEL+ cells / mm2
***P=1×10-14
***P=1×10-14
g
CD45
CD45/DAPI
100 μm
h
Relative mRNA levels
Tnf
Ccl2
Il1b
Il6

Supplementary Figure 4. Genetic inhibition of RIPK1 in hepatic UGDH-deficient mice does not reduce hepatic steatosis and inflammation in CD-HFD-fed mice.

a, RIPK1 kinase-dead mutation does inhibit TNFα/CHX-induced apoptosis in hepatocytes. Primary hepatocytes with indicated genotype were pre-treated with CHX (1 μM) for 0.5 h followed by TNFα (10 ng/ml) for 12 h. Cell death was measured by SytoxGreen positivity assay, data are represented as mean ± s.e.m. of *n* = 3 biologically independent experiments. Unpaired two-tailed t-test. **b**-**e**, Mice (16-week-old) with indicated genotypes were fed with CD-HFD for 8 weeks. Body weight and liver weight of indicated mice (b). H&E and oil red O staining of liver sections of indicated mice (c). NAS score and lipid content of livers from indicated mice (d). Measurement of liver TG, TC and NEFA of indicated mice (e). *n* = 6 mice for each genotype (b-d). Mean ± s.e.m. (b, d, e). Unpaired two-tailed t-test (d, e). **f**, Inhibition of RIPK1 inhibits cell death in livers from CD-HFD-fed hepatic UGDH-deficient mice. Immunofluorescence images of TUNEL assay on liver sections from 16-week-old mice with indicated genotypes after feeding with CD-HFD for 8 weeks. Representative images out of *n* = 6 mice for each genotype are represented. Graph depicting numbers of $TUNEL^+$ cells on liver sections of indicated genotypes. Each dot represents an individual mouse. Mean ± s.e.m. One-way ANOVA, post hoc Dunnett's test. **g**, **h**, Hepatic UGDH deficiency does not affect macrophage infiltration and inflammation in livers of CD-HFD-induced NASH. Immunofluorescence images of CD45 (g) and quantitative RT-PCR analysis of pro-inflammatory cytokines and chemokines (h) of livers from 16-week-old mice with indicated genotypes after feeding with CD-HFD for 8 weeks. Representative images out of *n* = 6 mice for each genotype are represented (g). *n* = 6 mice per genotype. Each dot represents an individual mouse. Mean ± s.e.m (h).

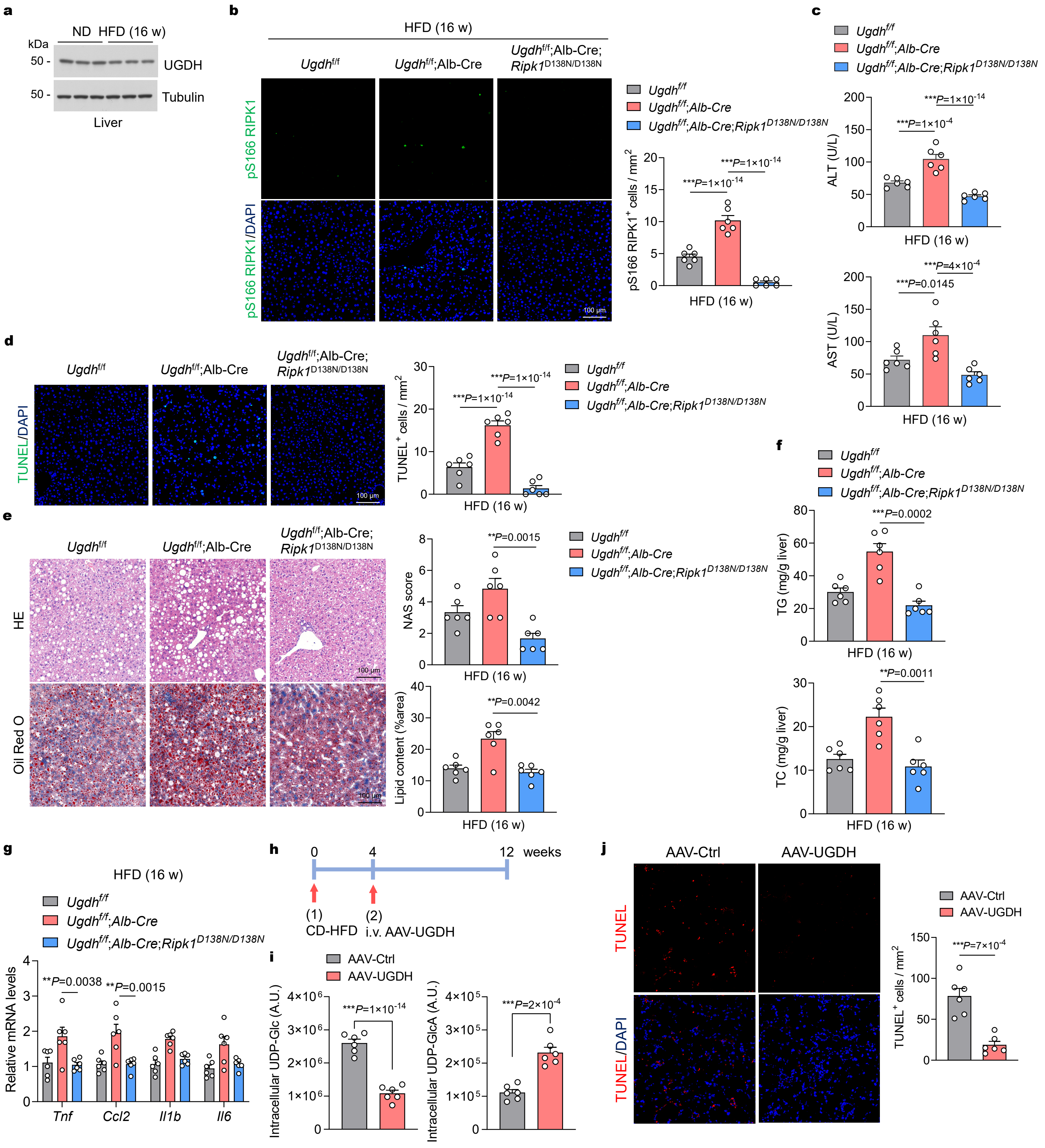
a
ND HFD (16 w)
kDa
50 -
UGDH
50 -
Tubulin
Liver
b
HFD (16 w)
Ugdh f/f
Ugdh f/f;Alb-Cre
Ugdh f/f;Alb-Cre; Ripk1 D138N/D138N
pS166 RIPK1
pS166 RIPK1/DAPI
100 μm
pS166 RIPK1+ cells / mm2
***P=1×10-14
***P=1×10-14
HFD (16 w)
c
ALT (U/L)
***P=1×10-4
***P=1×10-14
AST (U/L)
***P=0.0145
***P=4×10-4
HFD (16 w)
d
TUNEL/DAPI
TUNEL+ cells / mm2
***P=1×10-14
***P=1×10-14
e
HE
Oil Red O
NAS score
**P=0.0015
Lipid content (%area)
**P=0.0042
f
TG (mg/g liver)
***P=0.0002
TC (mg/g liver)
**P=0.0011
g
HFD (16 w)
Relative mRNA levels
**P=0.0038
**P=0.0015
Tnf Ccl2 Il1b Il6
h
0 4 12 weeks
(1) CD-HFD
(2) i.v. AAV-UGDH
i
AAV-Ctrl
AAV-UGDH
Intracellular UDP-Glc (A.U.)
***P=1×10-14
Intracellular UDP-GlcA (A.U.)
***P=2×10-4
j
AAV-Ctrl AAV-UGDH
TUNEL
TUNEL/DAPI
TUNEL+ cells / mm2
***P=7×10-4

Supplementary Figure 5. Genetic inhibition of RIPK1 in hepatic UGDH-deficient mice reduces hepatic steatosis and inflammation in conventional HFD-fed mice.

a, UGDH expression is not affected in mice fed with a conventional HFD. Representative immunoblotting analysis of UGDH protein levels in the livers of 24-week-old mice fed ND or a conventional HFD for 16 weeks. *n* = 3 mice per group. **b**, Hepatic UGDH deficiency increases RIPK1 activation in livers of HFD-induced NAFLD. Immunofluorescence images of p-S166 RIPK1 of liver sections from 24-week-old mice with indicated genotypes after feeding with a HFD for 16 weeks. Representative images out of *n* = 6 mice for each genotype are represented. Graph depicting numbers of p-S166 $RIPK1^+$ cells on liver sections of indicated genotypes. Each dot represents an individual mouse. Mean ± s.e.m. Unpaired two-tailed t-test. **c**, Hepatic UGDH deficiency aggravates RIPK1-driven liver damage in HFD-induced NAFLD. Serum levels of ALT and AST of 24-week-old mice with indicated genotypes after feeding with a HFD for 16 weeks. *n* = 6 mice for each genotype. Each dot represents an individual mouse. Mean ± s.e.m. One-way ANOVA, post hoc Dunnett's test. **d**, Immunofluorescence images of TUNEL assay on liver sections from 24-week-old mice after feeding with ND or a HFD for 16 weeks. Representative images out of *n* = 6 mice for each group are represented. Graph depicting numbers of $TUNEL^+$ cells on liver sections of indicated groups. Each dot represents an individual mouse. Mean ± s.e.m. Unpaired two-tailed t-test. **e-g**, Genetic inhibition of RIPK1 in hepatic UGDH-deficient mice reduces hepatic steatosis and inflammation in HFD-fed mice. Mice (24-week-old) with indicated genotypes were fed with a HFD for 16 weeks. H&E and oil red O staining of liver sections of indicated mice (e). Measurement of liver TG and TC of indicated mice (f). Quantitative RT-PCR analysis of hepatic pro-inflammatory cytokines and chemokines of indicated mice (g). *n* = 6 mice for each genotype. Each dot represents an individual mouse. Mean ± s.e.m. Unpaired two-tailed t-test. **h**, Schematic diagram of experimental design. Hepatic UGDH-deficient mice (20-week-old) were fed with CD-HFD for 12 weeks. After 4 weeks of CD-HFD feeding, mice were intravenously injected with AAV8-UGDH or AAV8-Ctrl along with continuous CD-HFD feeding. **i**, Overexpressing UGDH increases intracellular UDP-GlcA levels in livers of CD-HFD-fed mice. Intracellular UDP-

Glc and UDP-GlcA concentrations were determined in livers of mice from (h). *n* = 6 mice for each group. Each dot represents an individual mouse. Mean ± s.e.m. Unpaired two-tailed t-test. **j**, Overexpressing UGDH inhibits cell death in livers from CD-HFD-fed mice. Immunofluorescence images of TUNEL assay on liver sections of mice from (h). Representative images out of *n* = 6 mice for each genotype are represented. Graph depicting numbers of $TUNEL^+$ cells on liver sections of indicated genotypes. Each dot represents an individual mouse. Mean ± s.e.m. Unpaired two-tailed t-test.

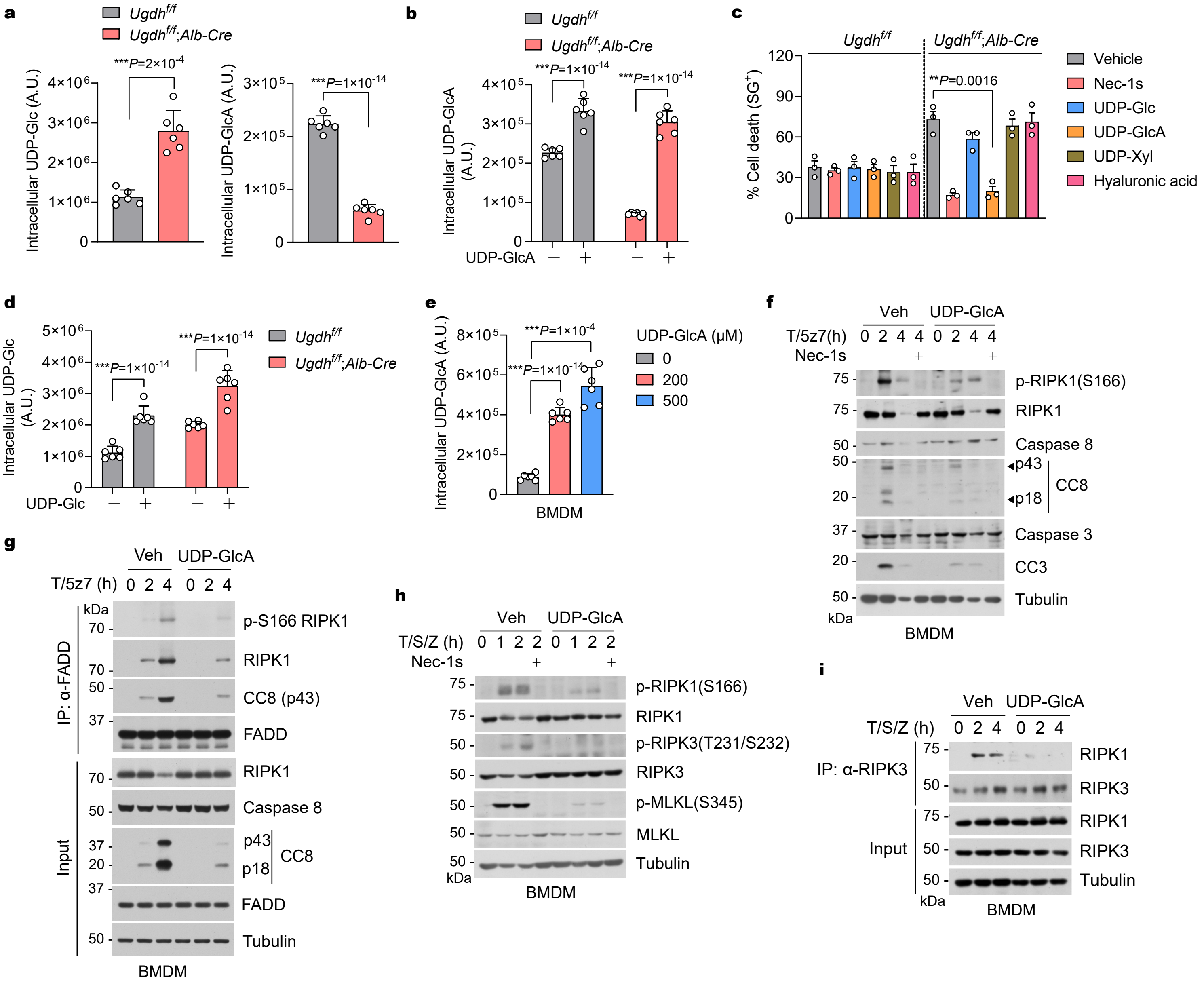
a
Ugdh f/f
Ugdh f/f;Alb-Cre
***P=2×10-4
Intracellular UDP-Glc (A.U.)
4×10 6
3×10 6
2×10 6
1×10 6
0
***P=1×10-14
Intracellular UDP-GlcA (A.U.)
3×10 5
2×10 5
1×10 5
0
b
Ugdh f/f
Ugdh f/f;Alb-Cre
***P=1×10-14
***P=1×10-14
Intracellular UDP-GlcA (A.U.)
4×10 5
3×10 5
2×10 5
1×10 5
0
UDP-GlcA – + – +
c
Ugdh f/f
Ugdh f/f;Alb-Cre
% Cell death (SG+)
120
90
60
30
0
**P=0.0016
Vehicle
Nec-1s
UDP-Glc
UDP-GlcA
UDP-Xyl
Hyaluronic acid
d
Intracellular UDP-Glc (A.U.)
5×10 6
4×10 6
3×10 6
2×10 6
1×10 6
0
***P=1×10-14
***P=1×10-14
Ugdh f/f
Ugdh f/f;Alb-Cre
UDP-Glc – + – +
e
Intracellular UDP-GlcA (A.U.)
8×10 5
6×10 5
4×10 5
2×10 5
0
***P=1×10-4
***P=1×10-14
UDP-GlcA (μM)
0
200
500
BMDM
f
Veh UDP-GlcA
T/5z7(h) 0 2 4 4 0 2 4 4
Nec-1s + +
75 p-RIPK1(S166)
75 RIPK1
50 Caspase 8
50 p43
20 p18
CC8
37 Caspase 3
20 CC3
50 Tubulin
kDa
BMDM
g
Veh UDP-GlcA
T/5z7 (h) 0 2 4 0 2 4
kDa
70 p-S166 RIPK1
70 RIPK1
50 CC8 (p43)
37 FADD
IP: α-FADD
70 RIPK1
50 Caspase 8
37 p43
20 p18
CC8
37 FADD
50 Tubulin
Input
BMDM
h
Veh UDP-GlcA
T/S/Z (h) 0 1 2 2 0 1 2 2
Nec-1s + +
75 p-RIPK1(S166)
75 RIPK1
50 p-RIPK3(T231/S232)
50 RIPK3
50 p-MLKL(S345)
50 MLKL
50 Tubulin
kDa
BMDM
i
Veh UDP-GlcA
T/S/Z (h) 0 2 4 0 2 4
IP: α-RIPK3
75 RIPK1
50 RIPK3
Input
75 RIPK1
50 RIPK3
50 Tubulin
kDa
BMDM

Supplementary Figure 6. UDP-GlcUA inhibits RIPK1-dependent apoptosis and necroptosis. **a**, Hepatic deletion of UGDH decreases intracellular UDP-GlcA levels in livers. Intracellular UDP-Glc and UDP-GlcA concentrations were determined in livers of mice fed with chow diet for 16 weeks. *n* = 6 mice for each group. Each dot represents an individual mouse. Mean ± s.e.m. Unpaired two-tailed t-test. **b**, Hepatocytes with indicated genotypes were supplemented with or without UDP-GlcA (0.5 mM) for 12 h, intracellular UDP-GlcA concentrations were then determined. *n* = 6 independent samples for each group. Mean ± s.e.m. Two-way ANOVA, post hoc Bonferroni's test. **c**, Flox and UGDH-deficient hepatocytes were pre-treated with CHX (1 μM) for 0.5 h followed by TNFα (10 ng/ml) in the presence or absence of Nec-1s (10 μM), UDP-Glc (0.5 mM), UDP-GlcA (0.5 mM), UDP-Xyl (0.5 mM), and hyaluronic acid (50 μg/ml) for 24 h. Cell death was measured by SytoxGreen positivity assay, data are represented as mean ± s.e.m. of *n* = 3 biologically independent experiments. One-way ANOVA, post hoc Dunnett's test. **d**, Hepatocytes with indicated genotypes were supplemented with or without UDP-Glc (0.5 mM) for 12 h, intracellular UDP-Glc concentrations were then determined. *n* = 6 independent samples for each group. Mean ± s.e.m. Two-way ANOVA, post hoc Bonferroni's test. **e**, UDP-GlcA supplementation increases its intracellular levels in BMDM cells. BMDMs were supplemented with or without UDP-GlcA with indicated concentrations for 12 h, intracellular UDP-GlcA concentrations were then determined. *n* = 6 independent samples for each group. Mean ± s.e.m. One-way ANOVA, post hoc Dunnett's test. **f**, UDP-GlcA inhibits RIPK1 kinase-dependent apoptosis. BMDMs were pre-treated with 5z7 (100 nM) for 0.5 h followed by TNFα (1 ng/ml) for indicated time in the presence or absence of UDP-GlcA (0.2 mM). The levels of p-S166 RIPK1, CC8 and CC3 were determined by immunoblotting, similar results were obtained from *n* = 3 independent experiments. **g**, UDP-GlcA inhibits the formation of complex II. BMDMs were pre-treated with 5z7 (100 nM) for 0.5 h followed by TNFα (1 ng/ml) for indicated time in the presence or absence of UDP-GlcA (0.2 mM). The complex II was isolated by immunoprecipitation of FADD, RIPK1 and Caspase 8 binding was revealed by immunoblotting. Similar results were obtained from *n* = 3 independent experiments. **h**, UDP-GlcA inhibits RIPK1 kinase-dependent necroptosis. BMDMs were pre-treated with SM-

164 (S, 100 nM) and zVAD (10 μM) for 0.5 h followed by TNFα (1 ng/ml) for indicated time in the presence or absence of UDP-GlcA (0.2 mM). The levels of p-S166 RIPK1, p-T231/S232 RIPK3 and p-S345 MLKL were determined by immunoblotting, similar results were obtained from *n* = 3 independent experiments. **i**, UDP-GlcA inhibits the formation of necrosome. BMDMs were pre-treated with SM-164 (S, 100 nM) and zVAD (10 μM) for 0.5 h followed by TNFα (1 ng/ml) for indicated time in the presence or absence of UDP-GlcA (0.2 mM). The necrosome was isolated by immunoprecipitation of RIPK3, RIPK1 binding was revealed by immunoblotting. Similar results were obtained from *n* = 3 independent experiments.

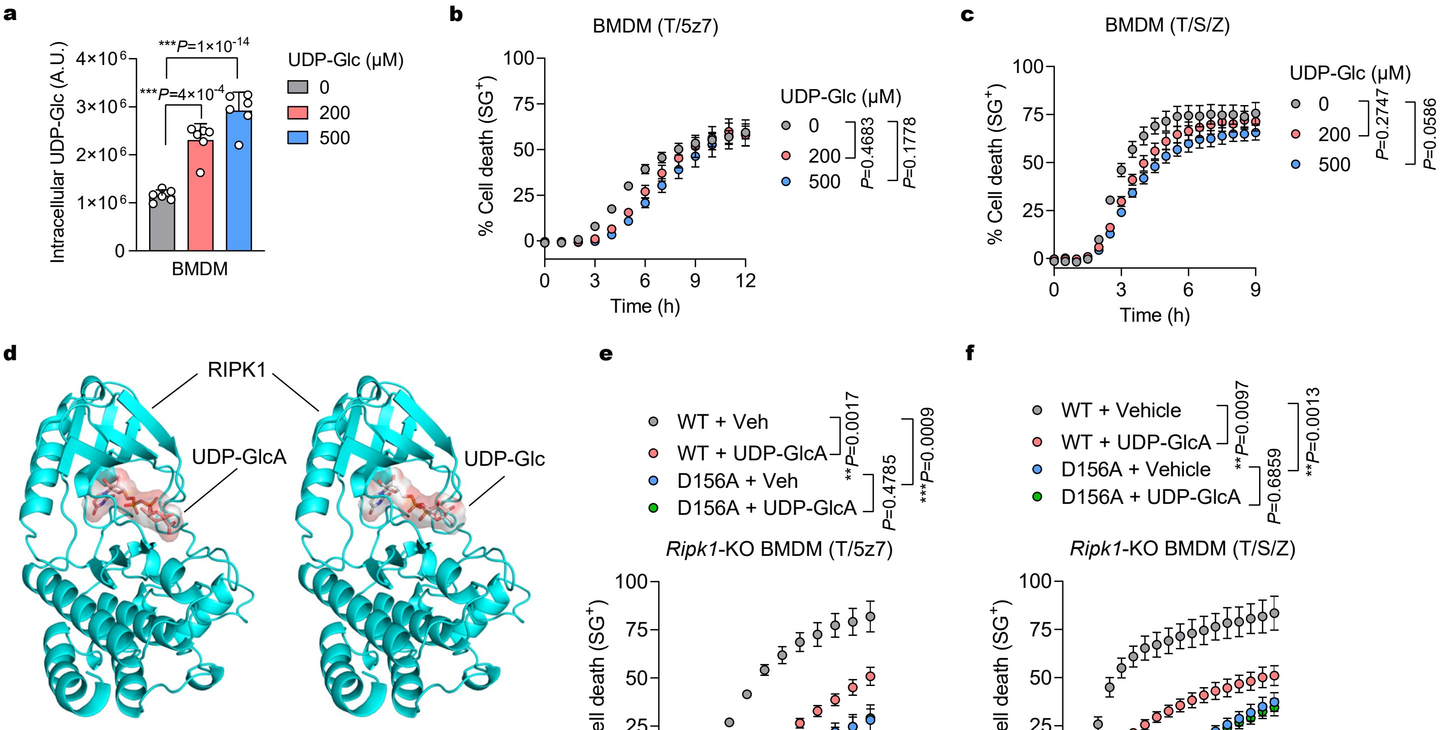

	UDP-GlcA	UDP-Glc
Binding free energy	Total	Total
	-60.3 kcal/mol	-39.5 kcal/mol

Supplementary Figure 7. UDP-GlcUA targets RIPK1.

a, UDP-Glc supplementation increases its intracellular levels in BMDM cells. BMDMs were supplemented with or without UDP-Glc with indicated concentrations for 12 h, intracellular UDP-Glc concentrations were then determined. n = 6 independent samples for each group. Mean ± s.e.m. One-way ANOVA, post hoc Dunnett's test. **b**, UDP-Glc does not inhibit RDA. BMDMs were pre-treated with 5z7 (100 nM) for 0.5 h followed by TNFα (1 ng/ml) for indicated time in the presence or absence of UDP-Glc with indicated concentration. Cell death was measured as a function of time by SytoxGreen positivity assay, data are represented as mean ± s.e.m. of n = 3 biologically independent experiments. Two-way ANOVA. **c**, UDP-Glc does not inhibit necroptosis. BMDMs were pre-treated with SM-164 (S, 100 nM) and zVAD (10 μM) for 0.5 h followed by TNFα (1 ng/ml) for indicated time in the presence or absence of UDP-Glc with indicated concentration. Cell death was measured as a function of time by SytoxGreen positivity assay, data are represented as mean ± s.e.m. of n = 3 biologically independent experiments. Two-way ANOVA. **d**, The binding of UDP-GlcA or UDP-Glc in complex with RIPK1-KD was generated by molecular docking. The protein was shown as cartoon and colored cyan. UDP-GlcA was shown as sticks. UDP-Glc and UDP-GlcA in the two ligand binding sites are all represented by a van der Waals surface, and the van der Waals surface are colored white and red. The binding free energy for UDP-GlcA and UDP-Glc with RIPK1-KD was shown below. **e**, **f**, WT and D156A RIPK1-reconstituted *Ripk1*-KO BMDMs were pre-treated with 5z7 (100 nM) (e) or SM-164 (S, 100 nM) and zVAD (10 μM) (f) for 0.5 h followed by TNFα (1 ng/ml) for indicated time in the presence or absence of UDP-GlcA (0.5 mM). Cell death was measured as a function of time by SytoxGreen positivity assay, data are represented as mean ± s.e.m. of n = 3 biologically independent experiments. Two-way ANOVA.

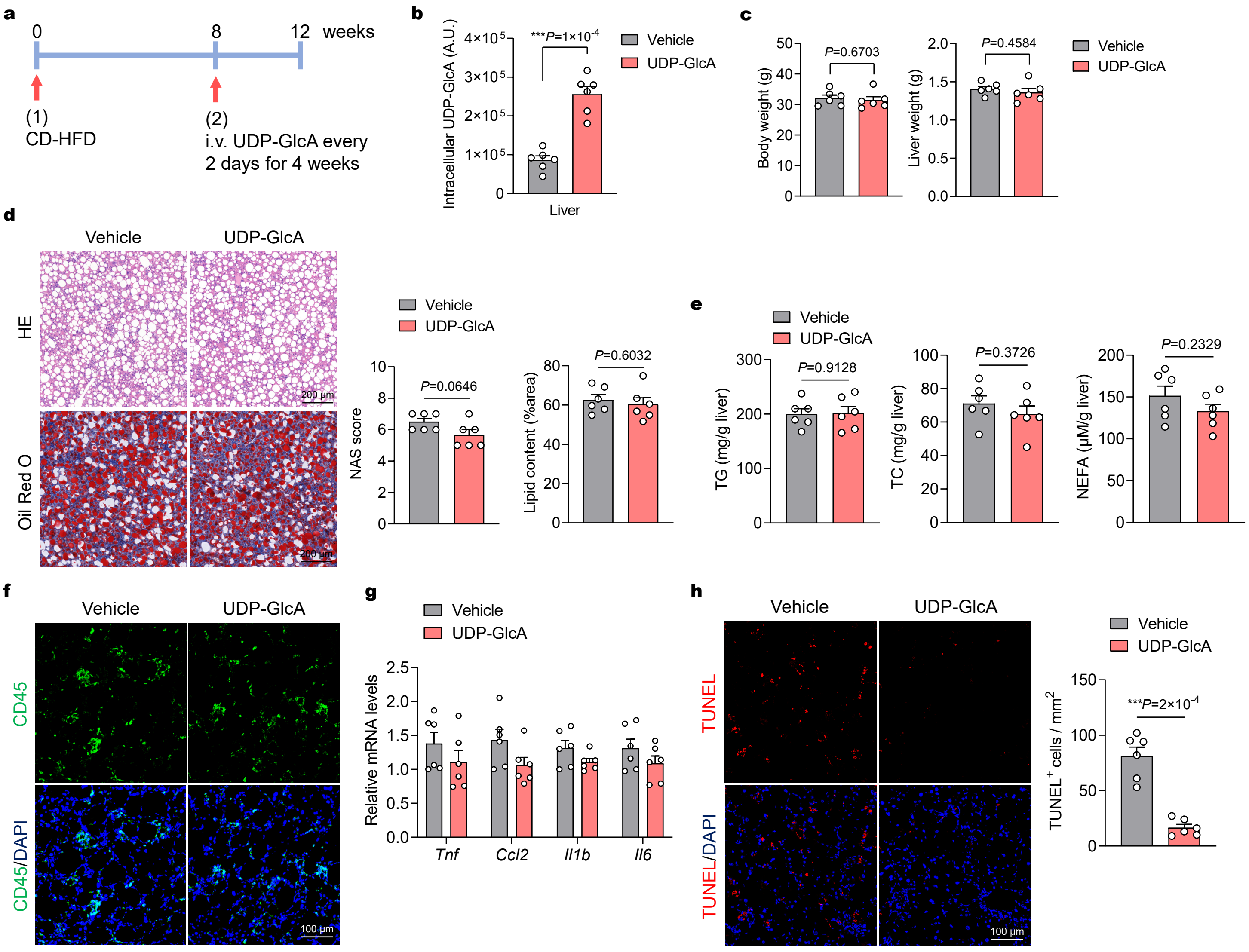
a
0
8
12 weeks
(1)
CD-HFD
(2)
i.v. UDP-GlcA every
2 days for 4 weeks
b
Intracellular UDP-GlcA (A.U.)
***P=1×10^-4
Liver
Vehicle
UDP-GlcA
c
Body weight (g)
P=0.6703
Liver weight (g)
P=0.4584
d
Vehicle
UDP-GlcA
HE
Oil Red O
200 μm
NAS score
P=0.0646
Lipid content (%area)
P=0.6032
e
TG (mg/g liver)
P=0.9128
TC (mg/g liver)
P=0.3726
NEFA (μM/g liver)
P=0.2329
f
CD45
CD45/DAPI
100 μm
g
Relative mRNA levels
Tnf
Ccl2
Il1b
Il6
h
TUNEL
TUNEL/DAPI
TUNEL+ cells / mm2
***P=2×10^-4

Supplementary Figure 8. UDP-GlcA reduces NASH-associated liver damage without affecting steatosis in CD-HFD-fed mice.

a, Schematic diagram of experimental design. WT mice (20-week-old) were fed with CD-HFD for 12 weeks. After 8 weeks of CD-HFD feeding, mice were intravenously injected with 200 mg/kg UDP-GlcA every two days for 4 weeks while fed continuous CD-HFD. **b**, UDP-GlcA administration increases in intracellular UDP-GlcA levels in livers of CD-HFD-fed mice. Intracellular UDP-GlcA concentrations were determined in livers of mice from (a). *n* = 6 mice for each group. Mean ± s.e.m. Unpaired two-tailed t-test. **c**, Body weight and liver weight of indicated mice from (a). Mean ± s.e.m. Unpaired two-tailed t-test. **d**, **e**, UDP-GlcA does not affect steatosis in CD-HFD-fed mice. Mice from (a) were subjected to H&E staining and Oil red O staining of liver sections (d), and measurement of liver TG, TC and NEFA (e). *n* = 6 mice for each group. Mean ± s.e.m. Unpaired two-tailed t-test. **f**, **g**, UDP-GlcA has minor effect on macrophage infiltration and inflammation in livers of CD-HFD-fed mice. Immunofluorescence images of CD45 (f) and quantitative RT-PCR analysis of pro-inflammatory cytokines and chemokines (g) of livers from mice that from (a). Representative images out of *n* = 6 mice for each genotype are represented (f). *n* = 6 mice per genotype. Each dot represents an individual mouse. Mean ± s.e.m (g). **h**, UDP-GlcA inhibits cell death in livers from CD-HFD-fed mice. Immunofluorescence images of TUNEL assay on liver sections of mice from (a). Representative images out of *n* = 6 mice for each group are represented. Graph depicting numbers of TUNEL$^+$ cells on liver sections of indicated genotypes. Each dot represents an individual mouse. Mean ± s.e.m. Unpaired two-tailed t-test.

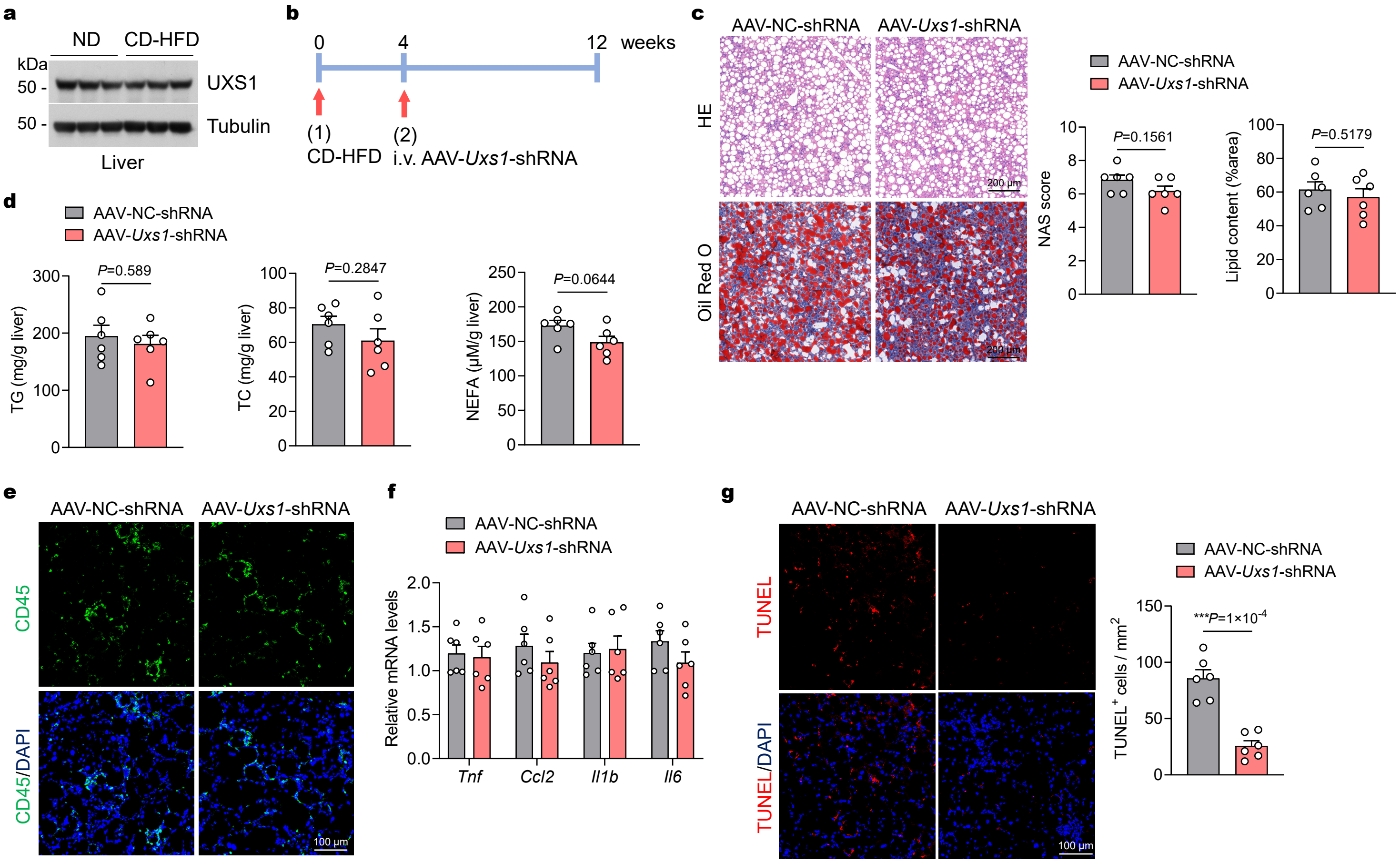
a
ND
CD-HFD
kDa
50 -
UXS1
50 -
Tubulin
Liver
b
0
4
12
weeks
(1)
CD-HFD
(2)
i.v. AAV-Uxs1-shRNA
c
AAV-NC-shRNA
AAV-Uxs1-shRNA
HE
Oil Red O
200 μm
NAS score
P=0.1561
Lipid content (%area)
P=0.5179
d
TG (mg/g liver)
P=0.589
TC (mg/g liver)
P=0.2847
NEFA (μM/g liver)
P=0.0644
e
CD45
CD45/DAPI
100 μm
f
Relative mRNA levels
Tnf
Ccl2
Il1b
Il6
g
TUNEL
TUNEL/DAPI
TUNEL+ cells / mm2
***P=1×10-4
100 μm

Supplementary Figure 9. Knockdown of UXS1 reduces NASH-associated liver damage without affecting steatosis in CD-HFD-fed mice.

a, UXS1 levels is not altered in CD-HFD induced NASH. Representative immunoblotting analysis of UXS1 protein levels in the livers of 20-week-old mice fed ND or CD-HFD for 12 weeks. *n* = 3 mice per group. **b**, Schematic diagram of experimental design. WT mice (20-week-old) were fed with CD-HFD for 12 weeks. After 4 weeks of CD-HFD feeding, mice were intravenously injected with AAV8-shRNA-*Uxs1* or AAV8-shRNA-NC along with continuous CD-HFD feeding. **c**, **d**, UXS1 knockdown does not affect steatosis in CD-HFD-fed mice. Mice from (b) were subjected to H&E staining and Oil red O staining of liver sections (c), and measurement of liver TG, TC and NEFA (d). *n* = 6 mice for each group. Mean ± s.e.m. Unpaired two-tailed t-test. **e**, **f**, Knockdown of UXS1 does not affect macrophage infiltration and inflammation in livers of CD-HFD-fed mice. Immunofluorescence images of CD45 (e) and quantitative RT-PCR analysis of pro-inflammatory cytokines and chemokines (f) of livers from mice that from (b). Representative images out of *n* = 6 mice for each genotype are represented (e). *n* = 6 mice per genotype. Each dot represents an individual mouse. Mean ± s.e.m (f). **g**, UXS1 knockdown inhibits cell death in livers from CD-HFD-fed mice. Immunofluorescence images of TUNEL assay on liver sections of mice from (b). Representative images out of *n* = 6 mice for each group are represented. Graph depicting numbers of TUNEL$^+$ cells on liver sections of indicated genotypes. Each dot represents an individual mouse. Mean ± s.e.m. Unpaired two-tailed t-test.

Patient Number	Steatosis grade	Lobular inflammation	Ballooning	Macrophage infiltration	Fibrosis	NAS	ALT(U/L)	AST(U/L)	Relative UGDH level
201803104	1	2	2	0	0	5	14.8	32.2	0.607
201804992	3	0	0	0	0	3	32.0	24.0	0.619
F201801843	0	0	2	0	0	2	14.9	21.4	0.883
F201802049	1	0	1	0	0	2	42.0	121.0	0.755
F201802374	1	0	0	0	0	1	28.0	37.0	0.983
F201803036	0	1	1	0	0	2	36.2	46.5	0.779
201809149	2	0	1	0	0	3	23.0	32.0	0.717
F201803581	0	1	1	0	0	2	150.0	52.0	0.684
F201805987	1	2	2	0	0	5	81.0	83.0	0.532
F201806562	1	0	1	0	0	2	29.0	23.0	0.834
201820134	1	0	0	0	0	1	48.0	58.0	0.944
F201807132	0	0	1	0	0	1	17.0	42.0	0.888
F201807914	1	0	0	0	0	1	17.0	57.9	0.898
201908005	0	3	0	0	0	3	40.0	132.0	0.648
F201903263	1	2	0	0	1	4	62.0	56.0	0.602
F201903059	0	1	0	0	0	1	19.0	26.0	1.079
201914098	0	0	0	0	3	3	35.0	80.0	0.698
F201905080	1	2	2	0	0	5	33.0	38.0	0.479
201917216	1	0	0	0	0	1	19.0	18.0	0.919
F202001140	1	0	2	0	0	3	74.0	72.0	0.618
202011313	1	1	2	0	0	4	42.0	34.0	0.511
202013813	1	0	2	0	0	3	16.0	53.0	0.593
202101529	1	2	2	0	1	6	50.0	55.0	0.307
202111556	1	3	2	0	0	6	92.8	221.2	0.488
202111682	1	1	2	0	0	4	87.0	105.0	0.545
F202106767	1	2	1	0	0	4	77.0	93.0	0.514
F202107631	1	2	2	1	1	7	105.0	95.0	0.311
F202107880	1	2	0	1	0	4	132.0	55.0	0.688
202108833	3	1	3	1	3	11	115.0	83.0	0.144
202004146	0	0	0	0	0	0	10.0	14.0	1.000
F202001725	0	0	0	0	0	0	13.0	18.0	1.146
F201804957	0	0	0	0	0	0	9.0	15.0	0.985
F201907051	0	0	0	0	0	0	31.0	9.0	1.086
F201907586	0	0	0	0	0	0	15.0	22.0	0.883
202011655	0	0	0	0	0	0	4.0	12.0	0.842

Supplementary Table 1

The clinical and histological characteristics of the human samples used in this study. These characteristics including steatosis grade, lobular inflammation, ballooning, macrophage infiltration, fibrosis, serum levels of ALT and AST.
