# Supplementary material for: UDP-glucuronate metabolism controls RIPK1-driven liver damage in nonalcoholic steatohepatitis
Source: Nat Commun. 2023 May 11;14:2715. doi: 10.1038/s41467-023-38371-2 (PMC10175487; doi:10.1038/s41467-023-38371-2)
Supplement: Supplementary file 3 — Reporting Summary [file 41467_2023_38371_MOESM3_ESM.pdf]

nature portfolio

Corresponding author(s): Jinyang Gu

Last updated by author(s): Apr 17, 2023

Reporting Summary

Nature Portfolio wishes to improve the reproducibility of the work that we publish. This form provides structure for consistency and transparency in reporting. For further information on Nature Portfolio policies, see our Editorial Policies and the Editorial Policy Checklist.

Statistics

For all statistical analyses, confirm that the following items are present in the figure legend, table legend, main text, or Methods section.

n/a	Confirmed	
☐	☒	The exact sample size (*n*) for each experimental group/condition, given as a discrete number and unit of measurement
☐	☒	A statement on whether measurements were taken from distinct samples or whether the same sample was measured repeatedly
☐	☒	The statistical test(s) used AND whether they are one- or two-sided<br>*Only common tests should be described solely by name; describe more complex techniques in the Methods section.*
☒	☐	A description of all covariates tested
☐	☒	A description of any assumptions or corrections, such as tests of normality and adjustment for multiple comparisons
☐	☒	A full description of the statistical parameters including central tendency (e.g. means) or other basic estimates (e.g. regression coefficient) AND variation (e.g. standard deviation) or associated estimates of uncertainty (e.g. confidence intervals)
☐	☒	For null hypothesis testing, the test statistic (e.g. *F*, *t*, *r*) with confidence intervals, effect sizes, degrees of freedom and *P* value noted<br>*Give P values as exact values whenever suitable.*
☒	☐	For Bayesian analysis, information on the choice of priors and Markov chain Monte Carlo settings
☒	☐	For hierarchical and complex designs, identification of the appropriate level for tests and full reporting of outcomes
☐	☒	Estimates of effect sizes (e.g. Cohen's *d*, Pearson's *r*), indicating how they were calculated

Our web collection on statistics for biologists contains articles on many of the points above.

Software and code

Policy information about availability of computer code

Data collection	Cell death assay: SYNERGY H1 microplate reader Gen5 software version 3.08.01 (BioTek)<br>qRT-PCR: QuantStudio 12K Flex software version 1.3 (Applied Biosystems)<br>Thermal shift assay: QuantStudio 12K Flex software version 1.3 (Applied Biosystems)<br>Images: Leica Application Suite X software version 1.8.1.13759 (Leica)<br>Gel blots scan: EPSON scan version 3.9.3.3SC (EPSON)
Data analysis	Results were analyzed using Prism version 8.4.1 (GraphPad Software).<br>Images and quantifications of immunoblots were analyzed by ImageJ version 1.52a.<br>Molecular modeling and docking were performed with the molecular simulation software suite Schrödinger (version 2018-1, Schrödinger, LLC, New York, NY, 2018).

For manuscripts utilizing custom algorithms or software that are central to the research but not yet described in published literature, software must be made available to editors and reviewers. We strongly encourage code deposition in a community repository (e.g. GitHub). See the Nature Portfolio guidelines for submitting code & software for further information.

Data

Policy information about availability of data

All manuscripts must include a data availability statement. This statement should provide the following information, where applicable:

- Accession codes, unique identifiers, or web links for publicly available datasets
- A description of any restrictions on data availability
- For clinical datasets or third party data, please ensure that the statement adheres to our policy

The crystal structure of RIPK1 kinase domain complexed with a high-affinity inhibitor was obtained from PDB (https://www.rcsb.org) with PDB code 7FD0.
The clinical and histological characteristics of the human samples are provided with this paper.
All other data generated or analyzed during this study are included in this published article and its supplementary information files.
Source data are provided with this paper.

Human research participants

Policy information about studies involving human research participants and Sex and Gender in Research.

Reporting on sex and gender	No sex and gender analyses were performed.
Population characteristics	31 male patients (age 47.4 ± 12.3) and 4 female patients (age 46.9 ± 12.6) with different stages of NAFLD were enrolled for this study. All the patients are Asian. The clinical and histological characteristics of the human samples are provided with this paper in Supplementary Table 1.
Recruitment	Steatotic livers were obtained from individuals with NAFLD or NASH who underwent liver biopsy or steatotic liver grafts. Liver steatosis due to excessive alcohol consumption (>140 g for men or >70 g for women, per week), use of toxins or drugs, and viral infection (for example, hepatitis B virus and hepatitis C virus) were excluded from the study. Samples from nonsteatotic liver were collected from normal donor livers that were allocated via China Organ Transplant Response System from 2017 to 2021. The donors were enrolled in the study on a volunteer basis, and the families of organ donors were approached for consent. Written informed consent was obtained from subjects or families of all participants. There was no self-selection bias or other biases that may be present and impact results.
Ethics oversight	All procedures involving human samples were approved by the Ethics Committee of Xinhua Hospital affiliated to Shanghai Jiao Tong University School of Medicine (Approval No. XHEC-D-2022-237), and were consistent with the principles outlined in the Declaration of Helsinki.

Note that full information on the approval of the study protocol must also be provided in the manuscript.

Field-specific reporting

Please select the one below that is the best fit for your research. If you are not sure, read the appropriate sections before making your selection.

☒ Life sciences ☐ Behavioural & social sciences ☐ Ecological, evolutionary & environmental sciences

For a reference copy of the document with all sections, see nature.com/documents/nr-reporting-summary-flat.pdf

Life sciences study design

All studies must disclose on these points even when the disclosure is negative.

Sample size	For cell culture-based experiments, sample sizes were determined based on pilot experiments and previous experience with the models and the methods used in this study: PMID: 30146158. The sample sizes for animal-based experiments were chosen based on generally expected variations of metabolic parameters and typical sample sizes for metabolic studies documented in literature (PMID: 29227477). Sample numbers were well described in the Figure legends.
Data exclusions	No data were excluded from the analyses.
Replication	All the biological experiments were repeated, at least, three times and reproduced. Western blotting data were confirmed by at least three independent samples.
Randomization	For animal experiments, age-matched mice with different genotypes were randomly divided into different experimental groups. For cell culture experiments, cells with different genotypes or treatments were randomly divided into different experimental groups.
Blinding	For in vitro experiments, blinding was difficult to apply, since experiments were performed by the same investigator. However, all experiments were independently performed mutiple times to ensure careful interpretation of the results. For animal experiments, the

operators were not blinded to the genotyping information, because the operator mainly raised and genotyped these mice. However, the investigators were blinded to the genotyping information during data collection and analysis, included immunofluorescence experiments, histological staining experiments.

Reporting for specific materials, systems and methods

We require information from authors about some types of materials, experimental systems and methods used in many studies. Here, indicate whether each material, system or method listed is relevant to your study. If you are not sure if a list item applies to your research, read the appropriate section before selecting a response.

Materials & experimental systems

n/a	Involved in the study
☐	☒ Antibodies
☐	☒ Eukaryotic cell lines
☒	☐ Palaeontology and archaeology
☐	☒ Animals and other organisms
☒	☐ Clinical data
☒	☐ Dual use research of concern

Methods

n/a	Involved in the study
☒	☐ ChIP-seq
☒	☐ Flow cytometry
☒	☐ MRI-based neuroimaging

Antibodies

Antibodies used

p-S166 RIPK1 (Biolynx, BX60008, 1:1000 for WB, 1:500 for IF)
RIPK1 (CST, 3493, 1:1000 for WB)
TNFR1 (CST, 13377, 1:1000 for WB)
cleaved Caspase-3 (CST, 9661, 1:1000 for WB, 1:400 for IF)
cleaved Caspase-8 (CST, 9429, 1:1000for WB)
RIPK3 (CST, 95702, 1:1000f or WB, 1:500 for IP)
p-T231/S232 RIPK3 (CST, 91702, 1:1000 for WB)
MLKL (CST, 37705, 1:1000 for WB)
p-S345 MLKL (Abcam, ab196436, 1:1000 for WB)
FADD (Abcam, ab124812, 1:1000 for WB)
FADD (Santa Cruz, SC-6036, 1:1000 for IP)
Caspase-3 (Proteintech, 19677-1-AP, 1:1000 for WB)
Caspase-8 (Proteintech, 13423-1-AP, 1:1000 for WB)
UGDH (Proteintech, 13151-1-AP, 1:1000 for WB)
UXS1 (Invitrogen, PA5-31629, 1:1000 for WB)
β-Tubulin (TRANS, HC101-02, 1:10000 for WB)
Alexa Fluor 488 goat anti-rabbit IgG (Invitrogen, A11034, 1:2000 for IF)
Alexa Fluor 568 goat anti-rabbit IgG (Invitrogen, A11011, 1:2000 for IF)
CD45 (Servicebio, GB11066, 1:500 for IF)

Validation

p-S166 RIPK1 Biolynx Cat#BX60008 YJY-1-5
http://www.biolynxtec.com/products/antibody/p-ripk1s166.html
Species specificity: Mouse
Applications: WB, IP, IF/ICC, IHC-Fr
Publications: PMID: 30146158

RIPK1 Cell Signaling Technology Cat#3493 D94C12
https://www.cellsignal.cn/products/primary-antibodies/rip-d94c12-xp-rabbit-mab/3493?site-search-type=Products&N=4294956287&Ntt=3493&fromPage=plp&_requestid=1999609
Species specificity: Human, mouse, rat, hamster, monkey
Applications: WB, IP, IF, F
Publications: PMID: 35831301, 35658939, 35641486

TNFR1 Cell Signaling Technology Cat#13377 D317K
https://www.cellsignal.cn/products/primary-antibodies/tnf-r1-d3i7k-rabbit-mab-rodent-specific/13377?site-search-type=Products&N=4294956287&Ntt=13377&fromPage=plp&_requestid=2005869
Species specificity: Mouse, rat
Applications: WB
Publications: PMID: 34376696, 33914027, 33152324

Cleaved Caspase-3 Cell Signaling Technology Cat#9661
https://www.cellsignal.cn/products/primary-antibodies/cleaved-caspase-3-asp175-antibody/9661?site-search-type=Products&N=4294956287&Ntt=9661&fromPage=plp&_requestid=2006946
Species specificity: Human, mouse, rat, monkey

Applications: WB, IP, IHC, IF, F
Publications: PMID: 35416106, 35894142, 35644004

cleaved Caspase-8 Cell Signaling Technology Cat#8592
https://www.cellsignal.cn/products/primary-antibodies/cleaved-caspase-8-asp387-d5b2-xp-rabbit-mab/8592?site-search-type=Products&N=4294956287&Ntt=cleavge+caspase-8&fromPage=plp
Species specificity: Mouse
Applications: WB, IP, IF, F
Publications: PMID: 36071040, 35859176, 35666872

RIPK3 Cell Signaling Technology Cat#95702 D4G2A
https://www.cellsignal.cn/products/primary-antibodies/rip3-d4g2a-rabbit-mab/95702?site-search-type=Products&N=4294956287&Ntt=95702&fromPage=plp&_requestid=2038936
Species specificity: Mouse
Applications: WB, IP, IF, F
Publications: PMID: 35873796, 35533409, 35711363

p-Thr231/Ser232 RIPK3 Cell Signaling Technology Cat#91702 E7S1R
https://www.cellsignal.cn/products/primary-antibodies/phospho-rip3-thr231-ser232-e7s1r-rabbit-mab/91702?site-search-type=Products&N=4294956287&Ntt=91702&fromPage=plp&_requestid=2039746
Species specificity: Mouse
Applications: WB, IF
Publications: PMID: 35533409, 35648750, 35587515

p-S345 MLKL Abcam Cat#ab196436 EPR9515(2)
https://www.abcam.cn/mlkl-phospho-s345-antibody-epr95152-ab196436.html
Species specificity: Mouse
Applications: WB, IP, Dot blot
Publications: PMID: 32999468, 33386754, 33431801

MLKL Cell Signaling Technology Cat#37705
https://www.cellsignal.cn/products/primary-antibodies/mlkl-d6w1k-rabbit-mab/37705?site-search-type=Products&N=4294956287&Ntt=37705&fromPage=plp&_requestid=1211819
Species specificity: Mouse
Applications: WB, IP
Publications: PMID: 32999468, 33386754, 33431801

FADD Abcam Cat#ab124812 EPR5030
https://www.abcam.cn/fadd-antibody-epr5030-ab124812.html
Species specificity: Mouse
Applications: Flow Cyt (Intra), WB, IHC-P, ICC, IP
Publications: PMID: 31949127, 32411316, 32428502

FADD Santa Cruz Cat#sc-6036
https://www.scbt.com/p/fadd-antibody-m-19?requestFrom=search
Species specificity: Mouse
Applications: IP
Publications: PMID: 24439895, 22362767, 21052097

Caspase-3 Proteintech Cat#19677-1-AP
https://www.ptgcn.com/products/CASP3-Antibody-19677-1-AP.htm
Species specificity: Human, mouse, rat
Applications: FC, IF, IHC, IP, WB, ELISA
Publications: PMID: 30568466, 35873644, 34570444

Caspase-8 Proteintech Cat#13423-1-AP
https://www.ptgcn.com/products/CASP8-Antibody-13423-1-AP.htm
Species specificity: Human, mouse
Applications: IF, IHC, IP, WB, ELISA
Publications: PMID: 36071040, 35859176, 35666872

UGDH Proteintech Cat#13151-1-AP
https://www.ptgcn.com/products/UGDH-Antibody-13151-1-AP.htm
Species specificity: Human, mouse, rat
Applications: IF, IHC, WB, ELISA
Publications: PMID: 31243371, 25465897, 25974208

UXS1 Invitrogen Cat#PA5-31629
https://www.thermofisher.cn/cn/zh/antibody/product/UXS1-Antibody-Polyclonal/PA5-31629

Species specificity: Human, mouse (Predicted reactivity: 100%)
Applications: IHC, WB
RRID: AB_2549102

β-Tubulin TRANS Cat#HC101-02
https://www.transgen.com.cn/antibody_reference/391.html
Species specificity: Human, mouse, rat
Applications: WB, ELISA, IF, IP
Publications: PMID: 28467790

Alexa Fluor 488 Invitrogen Cat#A11034
https://www.thermofisher.cn/cn/zh/antibody/product/Goat-anti-Rabbit-IgG-H-L-Highly-Cross-Adsorbed-Secondary-Antibody-Polyclonal/A-11034
Species specificity: Rabbit
Applications: ICC/IF, Flow
Publications: PMID: 36093836, 36250205, 36189424

Alexa Fluor 568 Invitrogen Cat# A-11011
https://www.thermofisher.cn/cn/zh/antibody/product/Goat-anti-Rabbit-IgG-H-L-Cross-Adsorbed-Secondary-Antibody-Polyclonal/A-11011
Species specificity: Rabbit
Applications: IHC (F), ICC/IF, Flow
Publications: PMID: 35662224, 36229429, 36229454

CD45 Servicebio Cat#GB11066
https://www.servicebio.cn/goodsdetail?id=1349
Species specificity: Mouse
Applications: IHC, IF
Publications: PMID: 32873321, 32512518, 29669342

Eukaryotic cell lines

Policy information about cell lines and Sex and Gender in Research

Cell line source(s)	Mouse primary hepatocytes and BMDMs were isolated by following a well-establised protocol and used in this study. HEK293T cells were purchased from ATCC (CRL-3216).
Authentication	All cell lines were authenticated in our lab by morphological examination using microscope and were not authenticated again genetically.
Mycoplasma contamination	The cells were tested by a TransDetect PCR Mycroplasma Detection Kit (Transgen Biotech, cat. no. FM311-01) to ensure that they are mycoplasma free.
Commonly misidentified lines (See ICLAC register)	There is no ICLAC line used in this study.

Animals and other research organisms

Policy information about studies involving animals; ARRIVE guidelines recommended for reporting animal research, and Sex and Gender in Research

Laboratory animals	·Male mice studied ranged between 6 to 20 weeks age.<br>·All mice were in the C57BL/6J background.<br>·Ugdh-flox/flox mice were from Cyagen, China (S-CKO-06534).<br>·Alb-Cre mice were from The Jackson Laboratory (Catalog No. 003574).<br>·Ripk1-D138N/D138N mice were generated as previous reported (PMID: 32513687).<br>·Ugdh-flox/flox;Alb-Cre mice were crossed with Ripk1-D138N/D138N mice to generate Ugdh-flox/flox;Alb-Cre;Ripk1-D138N/D138N mice.<br>·For choline-deficient high-fat-diet (CD-HFD)-induced NASH mouse model, 8-week-old male mice of indicated genotypes were fed CD-HFD (60% Fat, 0.1% Methionine and no added Choline, Research Diet, Cat. A06071302) or AMLN diet (40% Fat, 20% Fructose and 2% cholesterol, Research Diet, Cat. D09100301) for 8-12 weeks.<br>·For HFD-induced NAFLD mouse model, 8-week-old male mice of indicated genotypes were fed HFD (60% Fat, Research Diet, Cat. D12492) for 16 weeks.<br>·All animals were maintained in a specific pathogen-free environment and housed with no more than five animals per cage under controlled light (12-hour light and 12-hour dark cycle), temperature (24 ± 2oC) and humidity (50% ± 10%) conditions, and provided with ad libitum access to food and water throughout all experiments.
Wild animals	This study did not involve wild animals
Reporting on sex	To reduce the effect of sex variance, male mice aged 8 weeks were used for CD-HFD and HFD-induced NAFLD experiments.

Field-collected samples	This study did not involve samples collected from the field.
Ethics oversight	All animals were maintained in a specific pathogen-free environment, and animal experiments were conducted according to the protocols approved by the Standing Animal Care Committee at the Huazhong University of Science and Technology Tongji Medical College.

Note that full information on the approval of the study protocol must also be provided in the manuscript.
